# Automatic estimation of hallux valgus angle using deep neural network with axis-based annotation

**DOI:** 10.1007/s00256-024-04618-2

**Published:** 2024-03-13

**Authors:** Ryutaro Takeda, Hiroyasu Mizuhara, Akihiro Uchio, Toshiko Iidaka, Kenta Makabe, Taro Kasai, Yasunori Omata, Noriko Yoshimura, Sakae Tanaka, Takumi Matsumoto

**Affiliations:** 1 Department of Orthopaedic Surgery, Faculty of Medicine, The University of Tokyo, 7-3-1 Hongo, Bunkyo-Ku, Tokyo, 113-8655 Japan; 2 Department of Preventive Medicine for Locomotive Organ Disorders, 22Nd Century Medical and Research Center, Faculty of Medicine, The University of Tokyo, 7-3-1 Hongo, Bunkyo-Ku, Tokyo, 113-8655 Japan

**Keywords:** Deep learning, Hallux Valgus, Radiography, Foot

## Abstract

**Objectives:**

We developed the deep neural network (DNN) model to automatically measure hallux valgus angle (HVA) and intermetatarsal angle (IMA) on foot radiographs. The objective is to assess the accuracy of the model by comparing to the manual measurement of foot and ankle surgeons.

**Materials and methods:**

A DNN was developed to predict the bone axes of the first proximal phalanx and all metatarsals from the first to the fifth in foot radiographs. The dataset used for model development consisted of 1798 radiographs collected from a population-based cohort and patients at our foot and ankle clinic. The retrospective validation cohort comprised of 92 radiographs obtained from 92 consecutive patients visiting our foot and ankle clinic. The mean absolute error (MAE) between automatic measurements by the model and the median of manual measurements by three foot and ankle surgeons was compared to 3° using one-tailed *t*-test and was also compared to the inter-rater difference in manual measurements among the three surgeons using two-tailed paired *t*-test.

**Results:**

The MAE for HVA was 1.3° (upper limit of 95% CI 1.6°), and this was significantly smaller than the inter-rater difference of 2.0 ± 0.2° among the surgeons, demonstrating the superior accuracy of the model. In contrast, the MAE for IMA was 0.8° (upper limit of 95% CI 1.0°) that showed no significant difference from the inter-rater difference of 1.0 ± 0.1° among the surgeons.

**Conclusion:**

Our model demonstrated the ability to measure the HVA and IMA with an accuracy comparable to that of specialists.

**Supplementary Information:**

The online version contains supplementary material available at 10.1007/s00256-024-04618-2.

## Introduction

Hallux valgus is one of the most common forefoot deformities, characterized by a lateral deviation of the hallux and a medial protrusion of the first metatarsal head [1]. The severity of the hallux valgus deformity is commonly assessed using two radiographic indices: the hallux valgus angle (HVA) and the intermetatarsal angle (IMA) between the first and second metatarsals. HVA was defined as the angle between the bone axes of the proximal phalanx of the hallux (PH1) and the first metatarsal (MT1) on the dorsoplantar view in foot radiographs. The IMA is defined as the angle between the bone axes of MT1 and the second metatarsal (MT2) [2, 3]. These angles serve not only in diagnosing and assessing the severity of hallux valgus but also in guiding surgical procedure selection and evaluating the effectiveness of different surgical techniques [4–6].

Manual measurement methods for HVA and IMA were standardized by the ad hoc committee of the American Orthopedic Foot and Ankle Society (AOFAS) in 2002 [2, 3]. Although these standardized methods have proven to be highly reliable within 5°, the potential for bias and individual variability among different raters can still introduce challenges when comparing studies conducted by different evaluators.

Artificial intelligence including deep neural network (DNN) is one of a major breakthrough in translational medicine in orthopedic surgery [7]. Through the implementation of DNN for automated measurements, orthopedic surgeons can conduct bias-free assessments, enabling consistent and precise evaluations of hallux valgus severity across geographical boundaries. This standardized approach will foster a global interpretation of research outcomes related to hallux valgus, leading to advancements in both the understanding of the condition and improvements in its treatment. Although several previous studies have reported automatic measurement models for HVA, extracting the bone region details using DNN, these models have not achieved sufficient accuracy or undergone systematic validation [8–10]. An obstacle in automated measurements in foot radiographs is the potential overlap of bone areas, complicating the segmentation of individual bones, especially in cases of severe deformity. Another challenge arises from factors like spur formation, erosion, or joint dislocations affecting bone contours.

To address these challenges, we considered it advantageous to explore an approach that does not rely solely on the accurate extraction of bone regions. In this study, we adopted a novel method in which line segments representing bone axes drawn by a surgeon were directly used as annotation data. This innovative approach involved converting the drawn line segments into a probabilistic heatmap, enabling the training of the DNN.

The primary aim of this study was to develop a DNN capable of automatic bone axis estimation of PH1 and five metatarsals (MT1–5), thereby enabling the automatic measurement of the HVA and IMA. The secondary aim was to assess the accuracy of this model by comparing with the manual measurements.

## Materials and methods

### Model development

#### Datasets for model development

**Fig. 1:**
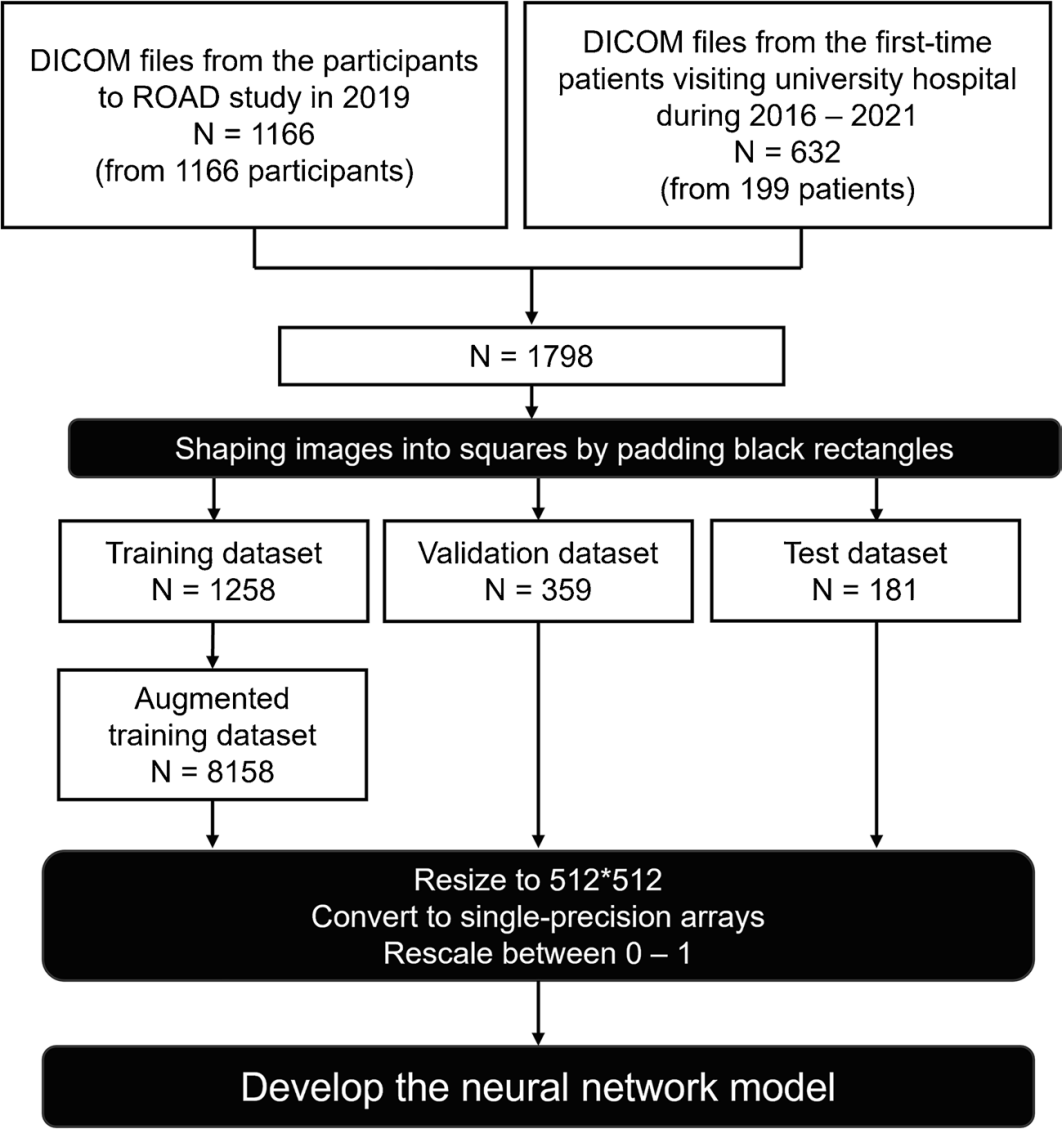
Datasets for model development

The data collection process is illustrated in Fig. 1. A total of 1798 dorsoplantar foot radiographs were obtained from two distinct populations. Of these, 1166 radiographs were obtained through the secondary use of data acquired in the resident cohort study known as Research on Osteoarthritis Against Disability (ROAD) study [11, 12]. All 1166 radiographs were obtained from bilateral imaging in a non-weightbearing condition, but only right-sided images were utilized. The remaining 632 radiographs were acquired from 199 patients who visited the foot and ankle department of our hospital between January 2016 and December 2021. These 632 radiographs were taken under weightbearing conditions and encompassed images of both the healthy and affected sides, as well as radiographs taken at various time points.

The 1798 radiographs collected were randomly assigned to three groups: a training dataset comprising 1258 radiographs, a validation dataset containing 359 radiographs, and a test dataset with 181 radiographs. The radiographs in the training dataset were augmented up to 6 times with random horizontal flips, and random rotations ranging from − 15° to 15°. To standardize the input for our deep learning model, the radiographs were adjusted by adding a black border for creating square images and resizing them to 512 × 512 pixels, preserving the original aspect ratio during the reduction. Subsequently, the pixel values were rescaled to fall within the range of 0 to 1.

#### Architecture of the model

The model had a U-net architecture, featuring five encoder-decoder blocks [13]. In this model, the input size was set to 512 × 512 pixels, and the output size was 512 × 512 × 6. This implies that the model generates 6-channel heatmaps, each representing a probabilistic area corresponding to the bone axes of PH1, MT1, MT2, MT3, MT4, and MT5. The output layer of the model contains a loss function known as root mean square error (RMSE).

#### Processing of annotation data

**Fig. 2:**
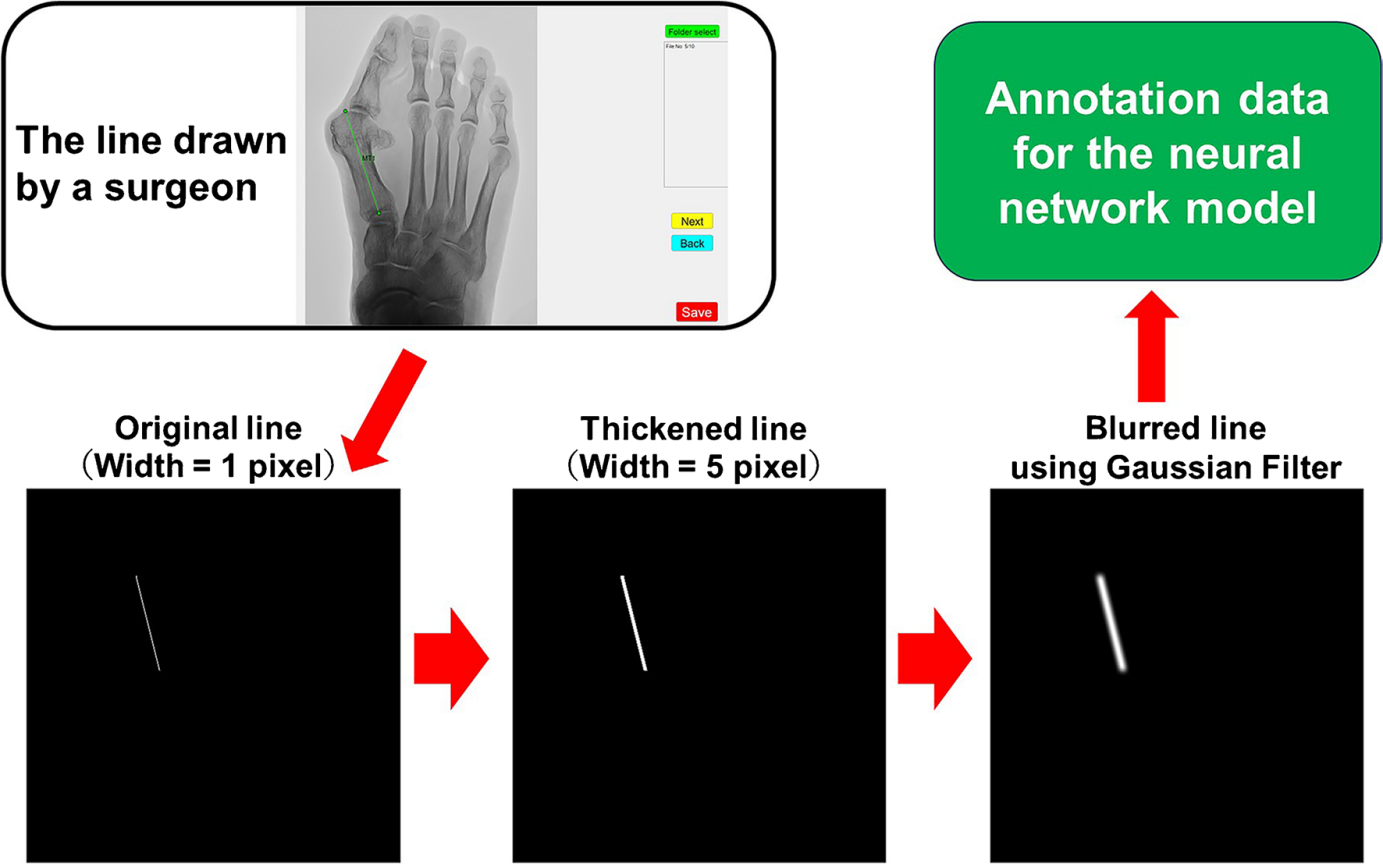
Flowchart illustrating the generation of the training annotation data for the neural network model from a line segment drawn by a surgeon

The process of handling the annotation data is outlined in Fig. 2. First, the bone axes of PH1, MT1, MT2, MT3, MT4, and MT5 were manually drawn by the author (R.T.), who is a board-certified specialist in orthopedic surgery, using a simple application developed specifically for this study. These drawings of the bone axes followed the recommendations of the AOFAS [2]. The endpoints of these line segments were placed at the end of the corresponding bone region along the bone axis. Next, the line segments were converted into 6-channel binary images in which the pixels situated beneath the lines were assigned a value of 1, whereas all other pixels were set to 0. To enhance the visibility and accuracy of these lines, the 1-pixel-wide lines were thickened to 5 pixels in width. Finally, a Gaussian filter was applied to the thickened lines to create a smoother representation. The standard deviation used in this Gaussian filter varied according to the specific bone. For the PH1 axis, the standard deviation was set to *h/2.5L*, while for MT1–MT5, it was set to *h/L* where *“h”* denotes the height of the image in pixels, and *“L”* denotes the length of the line segment.

#### Training and fine tuning of the neural network model

We utilized the Adam optimizer [14], for the training of our DNN. The maximal number of epochs was set to 12. Additionally, we fine-tuned other crucial parameters such as the minibatch size, initial learning rate, validation frequency, and validation patience. The Bayesian optimization algorithm was used in this process [15].

#### Angle calculations from the predicted model

**Fig. 3:**
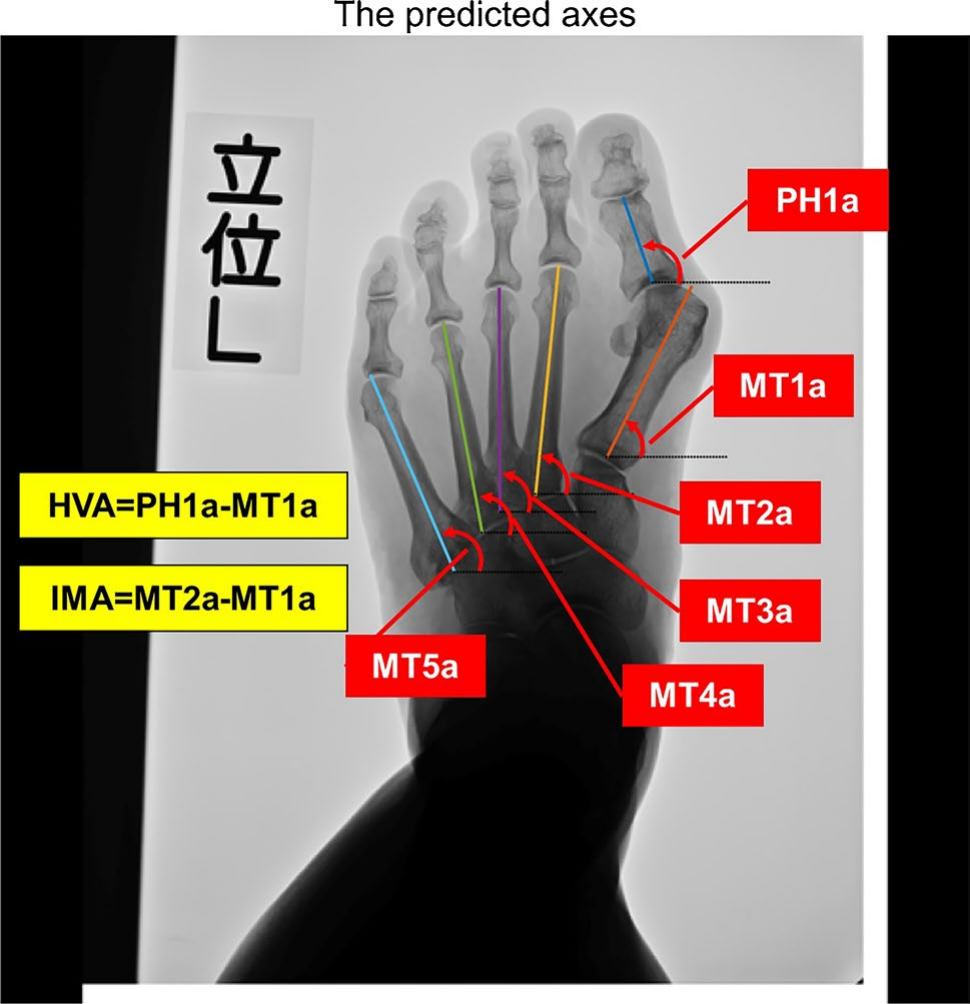
Predictions of the neural network model regarding the inclination angle of bone axes of the first proximal phalanx (PH1a), the first metatarsal (MT1a), the second metatarsal (MT2a), the third metatarsal (MT3a), the fourth metatarsal (MT4a), and the fifth metatarsal (MT5a). The hallux valgus angle (HVA) and intermetatarsal angle between the 1st and 2nd metatarsals (IMA) were calculated as the difference between PH1a and MT1a, and between MT2a and MT1a, respectively. In these angles, positive values indicated counterclockwise rotations, while negative values indicated clockwise rotations, with the right-facing horizontal line as the reference axis

Angle calculations were computed from the predicted model by employing linear regression of the high-value areas in the heatmaps. A more comprehensive explanation of this algorithm is described in Supplementary 1. From this process, the inclination angles of the PH1 and MT1–5 axes were obtained. These angles were used to calculate the HVA and IMA. Each angle is defined as either a positive or a negative value, as shown in Fig. 3.

### Retrospective validation cohort of the trained model

#### Dataset for the validation cohort

**Fig. 4:**
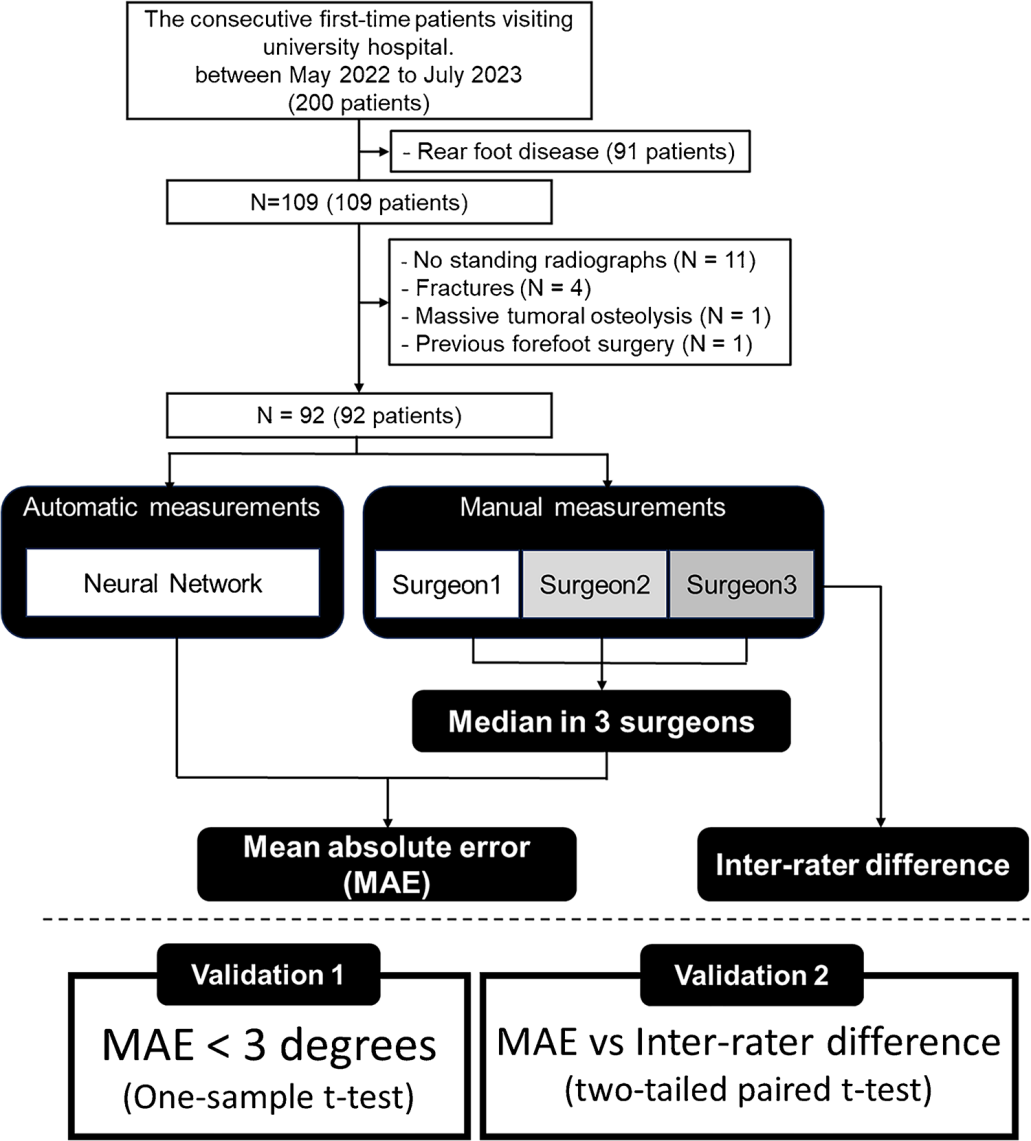
Validation workflow for comparing the automatic measurements made by the neural network model with the manual measurements made by foot and ankle surgeons

A database of 200 consecutive patients who firstly visited our foot and ankle clinic in the hospital between May 2022 and June 2023 was retrospectively reviewed for the validation of our model. We excluded the patients with rear-foot disease (91 patients), without weightbearing radiographs (11 patients), with foot fractures (4 patients), with massive tumoral osteolysis (1 patient), and with previous forefoot surgery (1 patient). Ultimately, 92 radiographs of 92 patients were included in the validation cohort (Fig. 4).

The minimal number of radiographs required for this study was estimated to be 92, using a *t*-test power analysis with a power of 0.99, to prove that the mean absolute error (MAE) was less than 3°, assuming that the MAE was 1.5° and the standard deviation (SD) of the error was 3.58°. Errors of less than 3° were considered good in a previous validation study for manual measurement by AOFAS [3]. An SD of 3.58° was derived from a previous study that attempted automatic HVA measurement [8].

#### Manual measurements by foot and ankle surgeons

Three foot and ankle surgeons with over 10 years of experience (rater 1 R.T., rater 2 T.K., and rater 3 A.U.) performed manual measurements for comparison with automatic measurements. Manual measurements were performed using the same software that was used to create the annotation data (Fig. 2). Each rater performed the measurements independently in a blinded manner.

#### Outcomes of validation

The primary outcome of validation was the MAE between manual and automatic measurements. A one-sample *t*-test was performed under the alternative hypothesis that the MAE was less than 3°. The MAE was calculated from the median of three manual measurements by foot and ankle surgeons. Additionally, the proportions of cases with errors of less than 3°, between 3° and 5°, and exceeding 5° were examined.

To evaluate the variation in manual measurements, we calculated the absolute differences in measurements between raters 1 and 2 (Diff_12_), 2 and 3 (Diff_23_), and 3 and 1 (Diff_31_). The interrater difference (Diff_123_) was defined as the mean of Diff_12_, Diff_23_, and Diff_31_. The MAE and Diff_123_ were compared using two-tailed paired *t*-tests. The significance level was set at* p* < 0.05 in the statistical tests.

### Software environment

All data processing and statistical analyses were performed using Deep Learning Toolbox in MATLAB 2022b (MathWorks Inc.). The code for the analysis and the trained model are available at https://data.mendeley.com/datasets/c24g4md953/1. In addition, a prototype Windows application is available on the same site that allows users to experience this model without coding.

### Ethics considerations

This study was approved by the Research Ethics Committee of The University of Tokyo Hospital (No. 1264, No. 2674–4). Written informed consent for the secondary use of data was obtained from all participants of the ROAD study. The informed consent from the patients who participated in this study was waived.

## Results

### Demographics of cases in the validation cohort

Demographic information for the 92 cases in the validation cohort, including age, sex, primary diagnosis, and manually measured HVA and IMA, is described in Table 1.

**Table 1:** Demographic data of the patients in the validation cohort

Age (years)	61.7 ± 17.4
Sex (male/female)	20/72
Patient side (right/left)	52/40
Diagnosis (number (%))	
Hallux valgus	46 (49%)
Adult acquired flat foot deformity	11 (12%)
Rheumatoid arthritis	10 (11%)
Hallux rigidus	7 (8%)
Plantar plate injury	4 (5%)
Sesamoid disorders	4 (5%)
Tumor	3 (5%)
Curly toe	2 (2%)
Psoriatic arthritis	2 (2%)
Others	3 (4%)
HVA in manual measurements (degrees)	29.9 ± 14.6
IMA in manual measurements (degrees)	14.3 ± 4.3

Age, HVA, and IMA are presented as mean ± standard deviation

The diagnosis of “Others” contains one case each of osteomyelitis, complex regional pain syndrome, and neuropathy of unknown cause

Abbreviations: *HVA*, hallux valgus angle; *IMA*, intermetatarsal angle between the first and second metatarsals

### Training and fine tuning of a neural network model

As a result of fine-tuning, the optimal DNN model was selected with a minibatch size of seven, an initial learning rate of 0.0001, a validation frequency of 200, and a validation patience of 10.

### Error analysis of automatic measurements

The results of the error analysis for automatic measurements are presented in Table 2. The MAE for HVA was 1.3° that was significantly lower than 3° (*p* < 0.01). Most cases (91%) had absolute errors of less than 3° between the manual and automatic measurements. Similarly, for the IMA, inclination angle of PH1, and inclination angle of MT1–5, the MAE values were all significantly less than 3°. Most of the cases had absolute errors of less than 3° between the manual and automatic measurements for these parameters.

**Table 2:** Absolute errors of automatic measurements compared to the median of manual measurements by three foot and ankle surgeons

Parameters	MAE	95% CI upper limit of MAE	*p-value (one-tailed t-test for MAE* < *3°)*	No. of cases (AE < 3°)	No. of cases (AE 3–5°)	No. of cases (AE > 5°)
HVA	1.3	1.6	< 0.01	84 (91%)	8 (9%)	2 (2%)
IMA	0.8	1.0	< 0.01	90 (98%)	2 (2%)	0 (0%)
PH1a	1.2	1.4	< 0.01	87 (95%)	5 (5%)	1 (1%)
MT1a	0.8	0.9	< 0.01	92 (100%)	0 (0%)	0 (0%)
MT2a	0.6	0.6	< 0.01	92 (100%)	0 (0%)	0 (0%)
MT3a	0.7	0.8	< 0.01	92 (100%)	0 (0%)	0 (0%)
MT4a	0.5	0.6	< 0.01	92 (100%)	0 (0%)	0 (0%)
MT5a	0.6	0.7	< 0.01	92 (100%)	0 (0%)	0 (0%)

*95% CI*, 95% confidence interval; *AE*, absolute error between automatic measurements and the median of manual measurements by three foot and ankle surgeons; *HVA*, hallux valgus angle; *IMA*, intermetatarsal angle between the first and second metatarsals; *MAE*, mean absolute error between automatic measurements and the median of manual measurements by three foot and ankle surgeons; *MT1-5a*, inclination angle of the axes of 1–5 metatarsals; *PH1a*, inclination angle of the axis of the first proximal phalanx

**Fig. 5:**
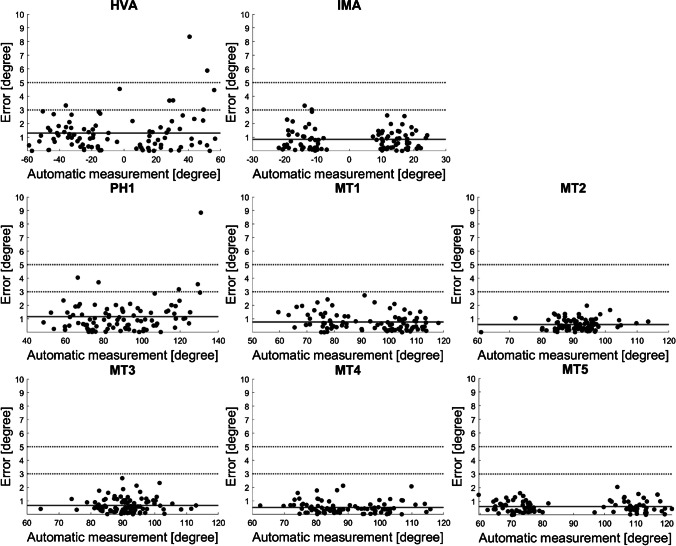
Scatter plots showing the distribution of absolute errors between automatic and manual measurements for various radiographic parameters. The *x*-axis displays the automatic measurement values for each radiographic parameter, while the *y*-axis represents the absolute error of the automatic measurements generated by the neural network model compared to the median of manual measurements for each parameter that were made by three foot and ankle surgeons. The dashed lines in the figures represent the boundaries for the absolute errors of 3° and 5°. Abbreviations: HVA, hallux valgus angle; IMA, intermetatarsal angle between the 1st and 2nd metatarsals; PH1, inclination angle of the axis of the first proximal phalanx; MT1–5, inclination angle of the axes of 1–5 metatarsals

Figure 5 shows the distribution of absolute errors from manual measurements by the surgeons for all 92 radiographs.

### Comparison of inter-rater differences and automatic measurement errors

The Diff_123_ for HVA, IMA, and the inclination angle of the axes of PH1 and MT1–5 were 2.0° ± 0.2°, 1.0° ± 0.1°, 1.6° ± 0.1°, 1.0° ± 0.2°, 0.5° ± 0.0°, 0.5° ± 0.0°, 0.7° ± 0.1°, and 0.8° ± 0.0°, respectively (mean ± SD). The MAE was significantly smaller than that of Diff_123_ in the HVA and inclination angles of the axes of PH1, MT1, MT4, and MT5 (*p* < 0.01, < 0.01, = 0.01, < 0.01, and < 0.01, respectively). No significant differences were observed in the IMA or inclination angle of the MT2 and MT3 axes (*p* = 0.15, 0.72, and 0.05, respectively).

### Visual presentation of automatic measurement cases

Visual representations of the predictions made by the developed neural DNN models for all 92 radiographs are presented in Supplementary 2.

**Fig. 6:**
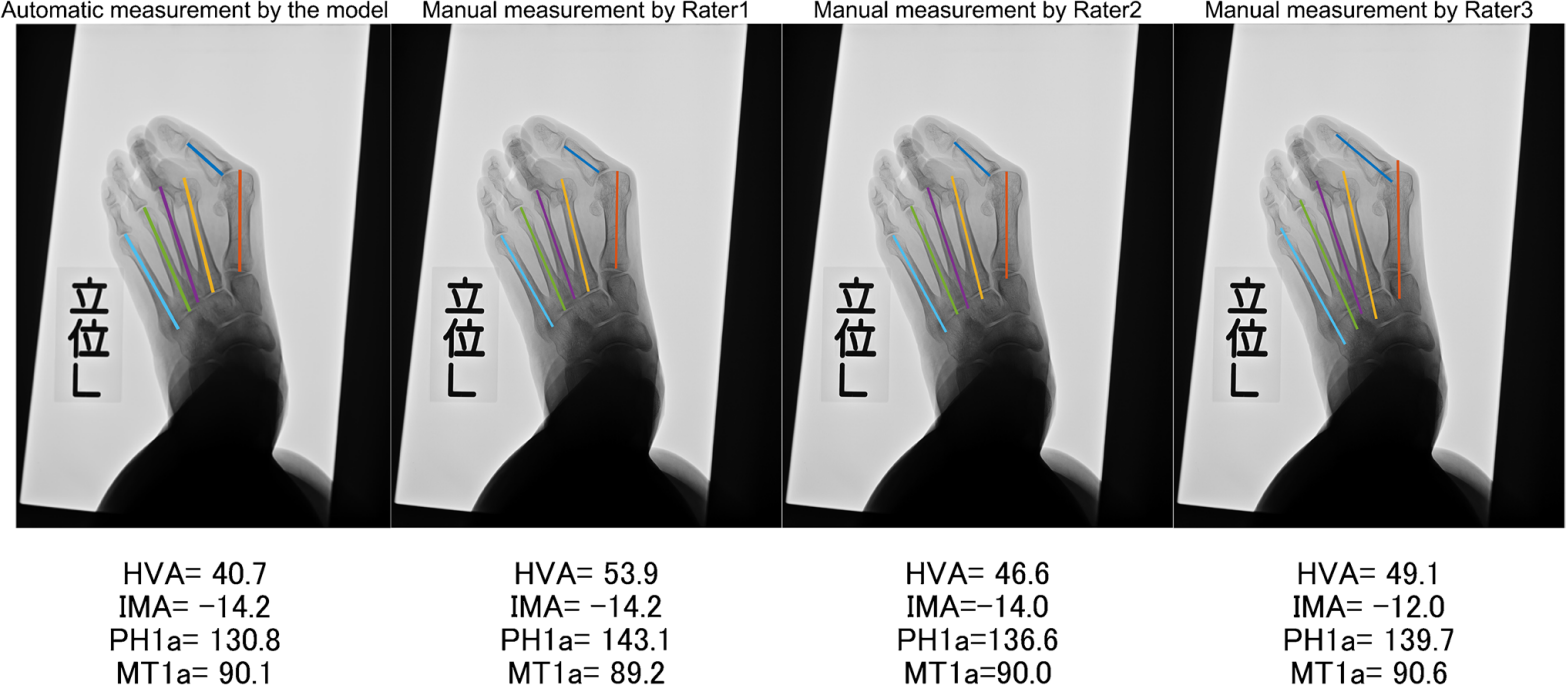
The largest absolute error in the hallux valgus angle measurement between automatic measurement obtained using the neutral network model and manual measurements, with an error of 8.3°. Abbreviations: HVA, hallux valgus angle; IMA, intermetatarsal angle; PH1a, inclination angle of the axis of the first proximal phalanx; MT1a, inclination angle of the axes of 1–5 metatarsals

The case with the worst error in the HVA is shown in Fig. 6. In this case, the error is 8.3°. Notably, Diff_123_ in this case was as high as 4.9°.

## Discussion

The current study describes the pioneering development of a DNN model designed for the automatic measurement of HVA and IMA. This model demonstrated accuracy comparable to manual measurements conducted by experienced foot and ankle surgeons.

The methods of manual measurement for HVA and IMA were established and assessed for reliability by the ad hoc committee of the AOFAS in 2002 [2, 3]. In a prior study, HVA was measured on 21 radiographs by 24 physicians with an error ranging from 3° (61.3% of physicians) to 5° (86.7% of physicians), averaged across 21 radiographs. In the current study, the MAE of the HVA and IMA were significantly smaller than 3°. Specifically, our model successfully measured the HVA and IMA with errors of less than 5° in 89–90 out of 92 cases (97–98%) and 92 of 92 cases (100%), respectively. In addition, the MAE of the HVA was significantly smaller than the inter-rater differences observed among the surgeons.

In the case with the most substantial error, with a deviation of 8.3° for the HVA, the inaccuracy can be attributed to an error in estimating the PH1 axis. This discrepancy may have arisen because of the notably high rotation of PH1 that compromised its symmetry, making accurate estimation challenging. Notably, in this case, the inter-rater differences in manual measurements by three surgeons were remarkably high at 4.9°, suggesting that even manual measurement faced challenges in accurately assessing this particular case.

Regarding the accuracy of the bone-axis estimation of MT1–5, the MAE was less than 1°, and no case exhibited errors exceeding 3°, indicating almost perfect accuracy.

Furthermore, it is important to consider the reproducibility of the measurements. In the reliability studies of manual measurements conducted by the AOFAS, as mentioned earlier, HVA was measured on 21 radiographs by 24 physicians over three sessions. The inter-session error ranged between 3° (61% of radiographs) and 5° (86.2% of radiographs), with results averaged across the 24 physicians [3]. In contrast, automated measurements by the DNN offer perfect reproducibility, a clear advantage over manual methods.

The first attempt to develop an automatic measurement of the HVA was reported in 2019 [10]. In that report, the entire bone area on foot radiographs was segmented using semantic segmentation with U-net. Subsequently, contours pertaining to the hallux were extracted by recognizing the medial protrusion of the bone contour. Finally, the bone axis and HVA were calculated from the midpoint of the hallux region. Another attempt was reported in 2020 [9], where the bone areas of PH1 and MT1 were identified by bounding box and subsequent thresholding. The linear regression line fitted to the midpoints of the segmented areas was predicted to be the bone axes of PH1 and MT1. It is worth noting that both of these previous methods face a fundamental challenge when bone areas overlap, rendering measurement virtually impossible. This limitation arises from the nature of segmentation that classifies each pixel in an image into specified region labels. Furthermore, neither of these earlier attempts systematically validated their accuracy.

The most recent attempt at automatic measurement, reported in 2022 [8], involved the development of a DNN capable of predicting anatomically characteristic points on bone contours using angle calculations based on the perpendicular bisector of these predicted points. The report concluded that anatomical point estimation had good accuracy; however, the HVA measurement still exhibited a substantial MAE of 5.22°. This may be attributable to the reliance of the angle calculation on points, making it the angle calculation sensitive to minor variations in bone contours. Furthermore, although the report conducted a validation using an independent dataset separate from the training dataset, cases with obvious variation in bone contours or joint disappearance were removed. By contrast, the current study employed a validation cohort that included patients with bone erosion, joint dislocation, or joint destruction caused by various diseases requiring forefoot surgery. This comprehensive approach allowed us to thoroughly evaluate accuracy and demonstrates a notable advancement in the field.

The high accuracy achieved by the current model is attributable to our innovative approach toward annotation data. Our method utilized heatmaps as annotation data to estimate line segments within the image. The technique using heatmaps was firstly proposed to estimate the key points in the object by Tompson et. al. in 2014 [16], and has since been applied successfully to detect anatomically distinct points in various contexts [8, 17, 18]. In the current study, we generated heatmaps from the line segment drawn by a specialist, and these were then used as annotation data. This approach enabled the ambiguous expression of the annotation data that usually varies even among the experts. Furthermore, converting entire line segments into heatmaps, rather than simply endpoints, improved prediction stability. This proved to be effective even in challenging scenarios involving bone erosion, dislocation, and overlapping bone areas.

Some limitations of our study must be acknowledged. One primary limitation is that the validation was conducted solely on radiographs from a single institute, introducing uncertainties about the model’s generalization performance. However, it is crucial to note that a significant portion of the radiographs used for training the DNN model were obtained from cohort studies, specifically in environments distinct from the hospital where the radiographs for validation were taken. We consider that the favorable results achieved under such circumstances serve as compelling evidence supporting the model’s generalization performance. A second limitation of the current model is its inapplicability to postoperative and post-trauma imaging. After trauma or postoperative interventions, defining the longitudinal axis of the bone becomes problematic due to significant structural changes and the inherent difficulty in establishing a clear reference point, making both precise manual measurements and accurate automated measurements challenging. In order to solve this confusion in defining the longitudinal axis of the first MT bone after the hallux valgus surgery, AOFAS recommends using the center of the metatarsal head as the distal reference point postoperatively, instead of the midpoint of the diaphysis which is used as the reference point preoperatively [2]. To develop a model applicable to postoperative radiographs, the model should be trained using annotation data according to the specific definitions for postoperative radiographs. Considering the diversity in hallux valgus surgery methods and implant types, it is necessary to collect postoperative images from multiple institutions. Future multicenter studies are expected to develop the automated measurement models for postoperative radiographs.

In conclusion, we successfully developed a DNN capable of accurately measuring the HVA, IMA, and inclination angles of the bone axes of PH1 and MT1–MT5. Our results demonstrate that measurements using this model closely replicate manual measurements by experienced foot and ankle surgeons. This advanced DNN holds great promise for future research involving foot radiographs, as it not only eliminates subjectivity, but also ensures complete reproducibility in repeated measurements. Its potential applications extend to various clinical and research settings and offer a reliable tool for assessing these critical angles.

## Supplementary Information

Below is the link to the electronic supplementary material.

Supplementary file1 (DOCX 380 KB)

Supplementary file2 (DOCX 1.35 MB)

## Data Availability

The data that support the findings of this study are available from the corresponding author, T.M., upon reasonable request.
