# Supplementary material for: Automatic estimation of hallux valgus angle using deep neural network with axis-based annotation
Source: Skeletal Radiol. 2024 Mar 13;53(11):2357–66. doi: 10.1007/s00256-024-04618-2 (PMC11410836; doi:10.1007/s00256-024-04618-2)
Supplement: Supplementary file 1 — Supplementary file1 (DOCX 380 KB) [file 256_2024_4618_MOESM1_ESM.docx]

Supplementary material 1

The algorism to draw a bone axis from the prediction

of the neural network model

1


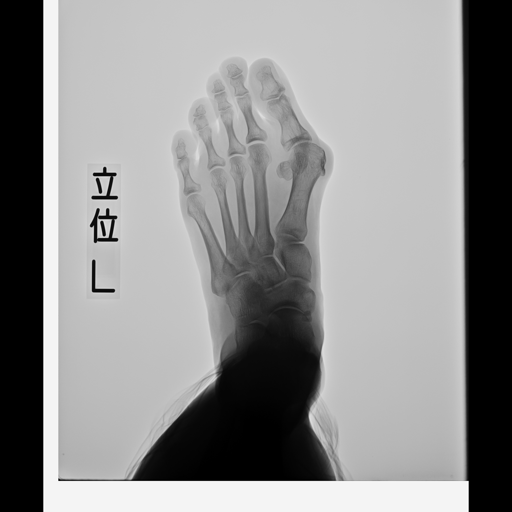

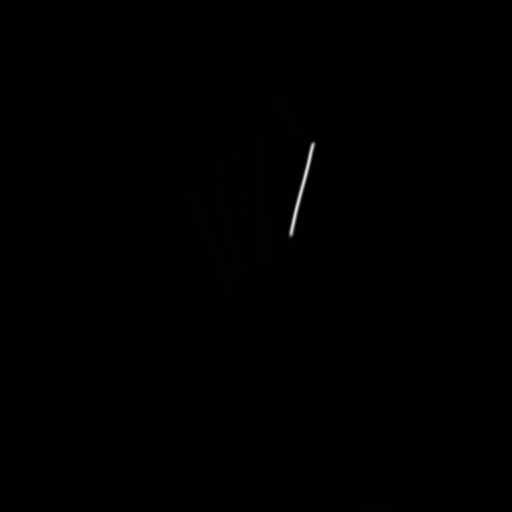


Heatmaps are predicted by the neural network model

2


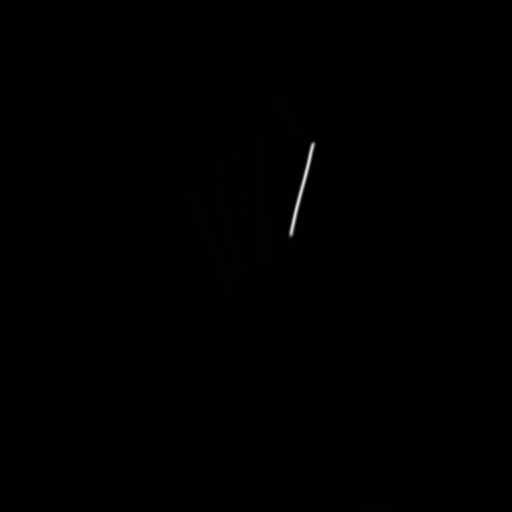

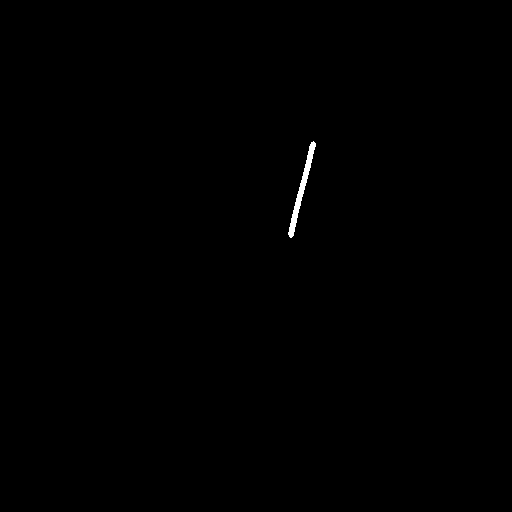


The pixels with which values are >0.1 are extracted. The binary mask image is obtained.

3


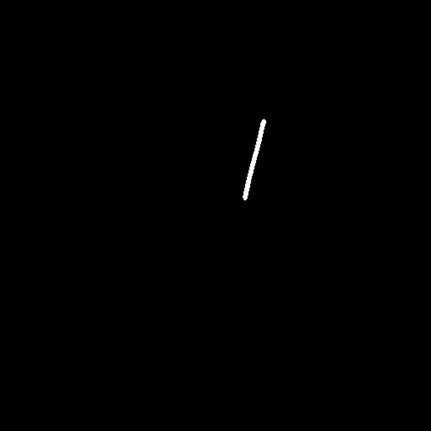

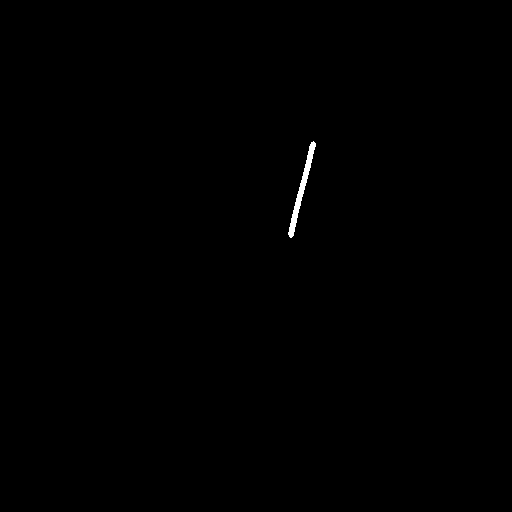


Holes in the white area are filled by morphology calculation.

The largest continuous area is extracted.

4


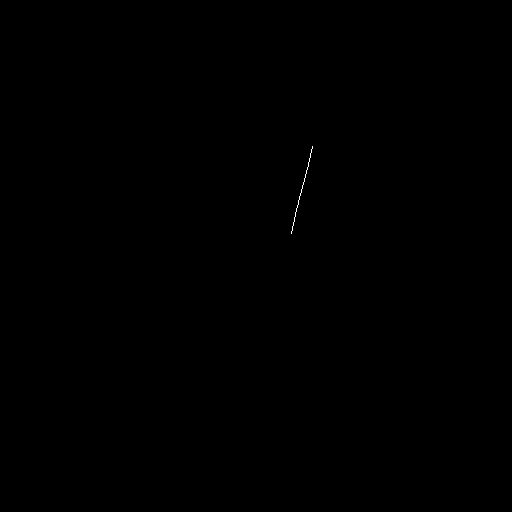

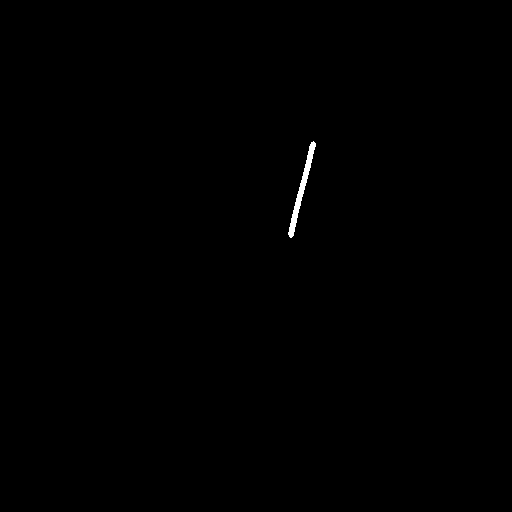


The skeleton of the area is obtained by morphological calculations.

5


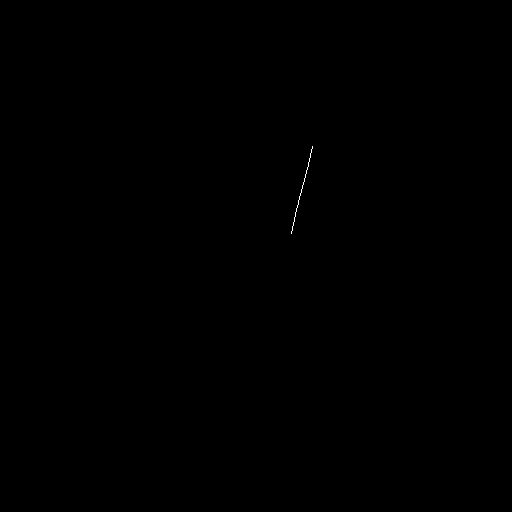

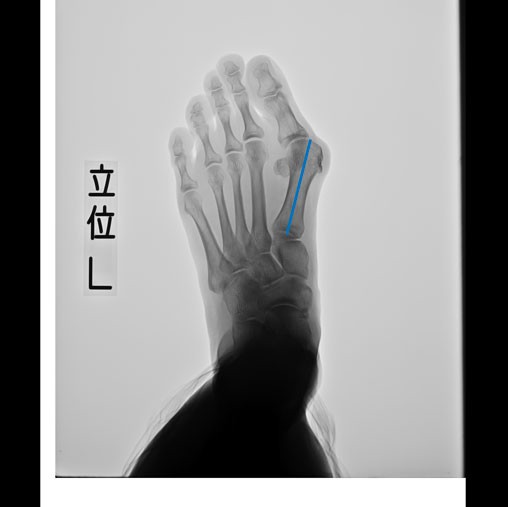


A linear regression line that fits the skeleton is drawn.

6
