# Supplementary material for: Automatic estimation of hallux valgus angle using deep neural network with axis-based annotation
Source: Skeletal Radiol. 2024 Mar 13;53(11):2357–66. doi: 10.1007/s00256-024-04618-2 (PMC11410836; doi:10.1007/s00256-024-04618-2)

Supplementary material 2

The prediction of bone axes by the deep neural network model on 92 radiographs.


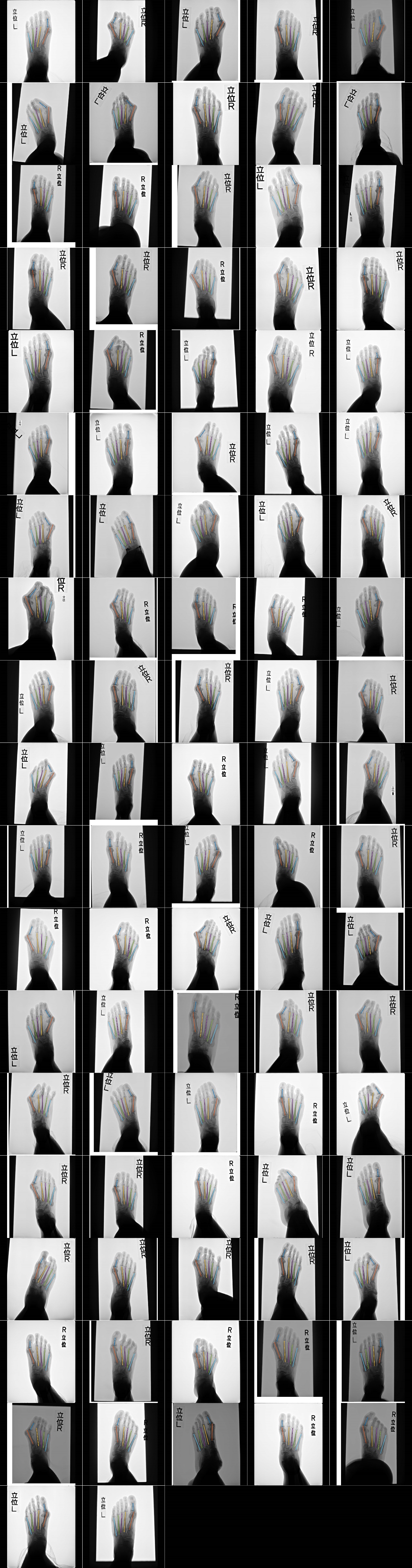

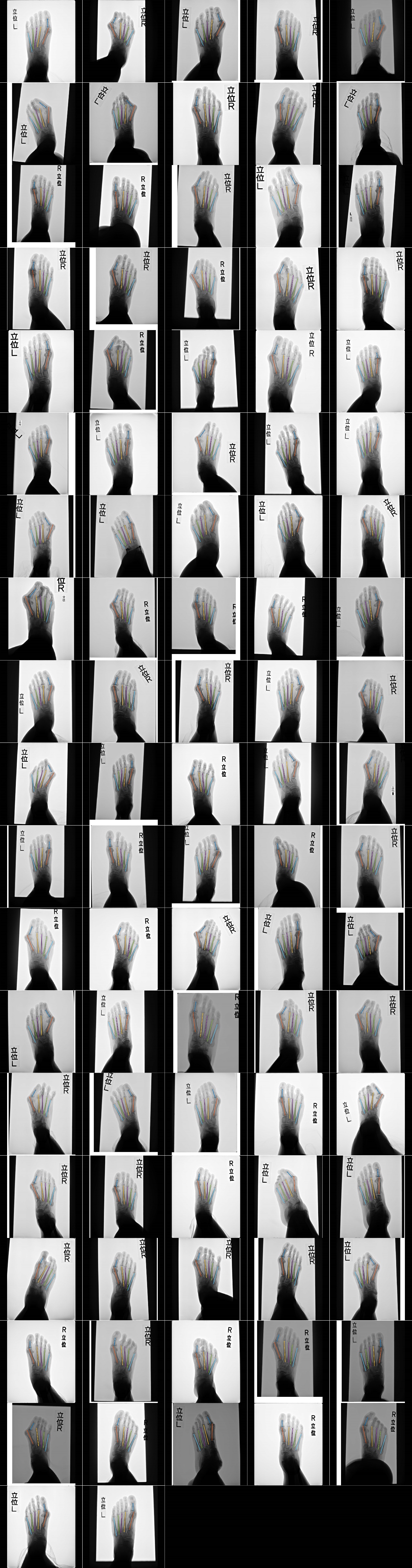


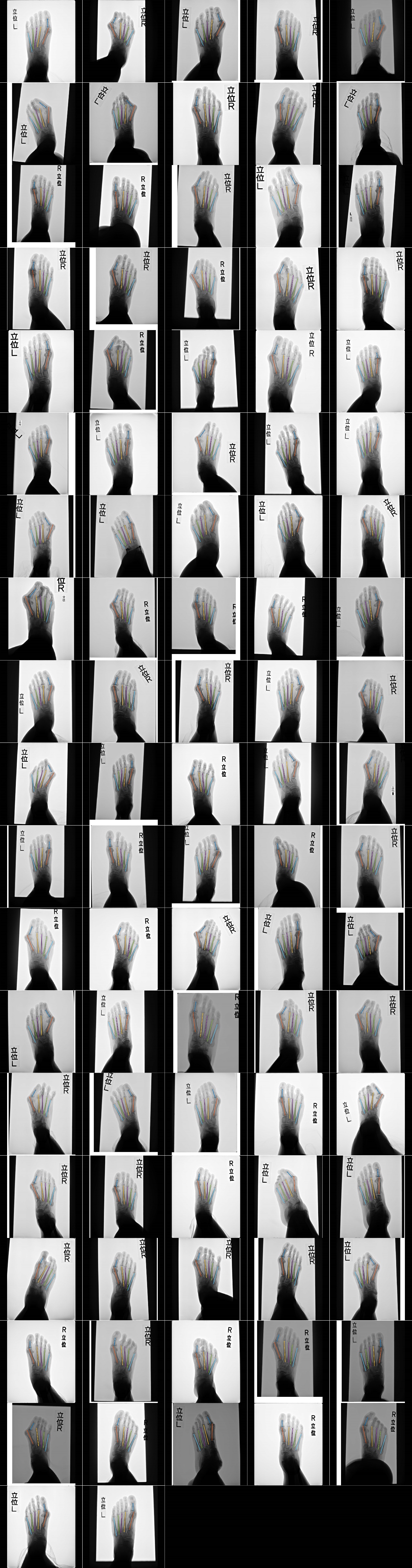

Supplement: Supplementary file 2 — Supplementary file2 (DOCX 1.35 MB) [file 256_2024_4618_MOESM2_ESM.docx]
